# Changes in Mobile Health Apps Usage Before and After the COVID-19 Outbreak in China: Semilongitudinal Survey

**DOI:** 10.2196/40552

**Published:** 2023-02-22

**Authors:** Le Yang, Jiadong Wu, Xiaoxiao Mo, Yaqin Chen, Shanshan Huang, Linlin Zhou, Jiaqi Dai, Linna Xie, Siyu Chen, Hao Shang, Beibei Rao, Bingtao Weng, Ayiguli Abulimiti, Siying Wu, Xiaoxu Xie

**Affiliations:** 1 School of Public Health Fujian Medical University Fuzhou China; 2 School of Nursing Fujian Medical University Fuzhou China

**Keywords:** application, China, COVID-19, mHealth, health management, mobile health, technology, app, survey, data, user, user experience, vaccination, download, healthcare, development

## Abstract

**Background:**

Mobile health (mHealth) apps are rapidly emerging technologies in China due to strictly controlled medical needs during the COVID-19 pandemic while continuing essential services for chronic diseases. However, there have been no large-scale, systematic efforts to evaluate relevant apps.

**Objective:**

We aim to provide a landscape of mHealth apps in China by describing and comparing digital health concerns before and after the COVID-19 outbreak, including mHealth app data flow and user experience, and analyze the impact of COVID-19 on mHealth apps.

**Methods:**

We conducted a semilongitudinal survey of 1593 mHealth apps to study the app data flow and clarify usage changes and influencing factors. We selected mHealth apps in app markets, web pages from the Baidu search engine, the 2018 top 100 hospitals with internet hospitals, and online shopping sites with apps that connect to smart devices. For user experience, we recruited residents from a community in southeastern China from October 2019 to November 2019 (before the outbreak) and from June 2020 to August 2020 (after the outbreak) comparing the attention of the population to apps. We also examined associations between app characteristics, functions, and outcomes at specific quantiles of distribution in download changes using quantile regression models.

**Results:**

Rehabilitation medical support was the top-ranked functionality, with a median 1.44 million downloads per app prepandemic and a median 2.74 million downloads per app postpandemic. Among the top 10 functions postpandemic, 4 were related to maternal and child health: pregnancy preparation (ranked second; fold change 4.13), women's health (ranked fifth; fold change 5.16), pregnancy (ranked sixth; fold change 5.78), and parenting (ranked tenth; fold change 4.03). Quantile regression models showed that rehabilitation (P_75_, P_90_), pregnancy preparation (P_90_), bodybuilding (P_50_, P_90_), and vaccination (P_75_) were positively associated with an increase in downloads after the outbreak. In the user experience survey, the attention given to health information (prepandemic: 249/375, 66.4%; postpandemic: 146/178, 82.0%; *P*=.006) steadily increased after the outbreak.

**Conclusions:**

mHealth apps are an effective health care approach gaining in popularity among the Chinese population following the COVID-19 outbreak. This research provides direction for subsequent mHealth app development and promotion in the postepidemic era, supporting medical model reformation in China as a reference, which may provide new avenues for designing and evaluating indirect public health interventions such as health education and health promotion.

## Introduction

### Background

In the wake of the COVID-19 outbreak, digital health technologies to assist medical service systems and people [1], such as telemedicine, have moved from a convenience to a demand [2-5]. Mobile health (mHealth) apps are a novel platform that uses mobile devices to acquire data across wellness and disease diagnosis, prevention, and management [6,7].

Before the COVID-19 outbreak, several studies investigated the characteristics of apps in China; these studies focused on only specific health domains, such as disease management, women's health, and sports, instead of elaborating on the overall mHealth app situation [8-11]. However, in the context of COVID-19, the development of mHealth apps has become a hot topic [12]. Only a few studies have investigated the mHealth app market status, focusing on the assessment of functional distribution but without refined classifications of mHealth apps and lacking information integrity [13]. Therefore, the classifications for mHealth apps have not been elaborated, and detailed descriptive research on mHealth apps is lacking.

After the outbreak, apps directly related to COVID-19 that were used to track high-risk groups and assist in diagnosis were the most studied [14-16]. One study provided an overview and classifications of mHealth apps currently available on the market to combat COVID-19, based on differences in basic functions and purpose [17]. However, in the face of the significant challenges posed by the pandemic, app types other than those related to COVID-19 were also considered valuable tools, such as easing the burden on hospitals, providing access to reliable information, tracking individuals’ symptoms and mental health, and discovering new predictors [18]. Previous studies have shown that mHealth apps can improve people's life needs (such as fertility), which promote their use [19]. However, the development trends for many types of mHealth apps have been complicated due to the pandemic, which requires attention.

At the same time, unlike other countries, China implemented normalized pandemic prevention and control and rarely used contact tracing apps in a personal form; thus, the apps developed directly as a result of the pandemic were not the focus of this research. There was a spike in the volume of phone calls asking medical questions after the outbreak with a great increase in online demand in China [17]. The unbalanced allocation of health resources between the east and west, due to the vast size of China, also led to the demand for telemedicine. During the COVID-19 pandemic, a multimodal telemedicine network in Sichuan Province in Western China was activated immediately, which was demonstrated to be feasible, acceptable, and effective [20]. Moreover, due to the unique national conditions of the 1-child policy and the aging population, changes in mHealth app fields before and after the outbreak are different from those in other countries [21,22]. Therefore, mHealth apps, as emerging tools, that focus on changes in various health functions before and after the outbreak are worth studying in China and could be important for prevention, diagnosis, treatment, and management decisions for future public health emergencies.

However, it is presently unclear what mHealth app characteristics, if any, have been influenced and appropriately deployed in the pre and postpandemic periods.

### Objectives

Here, we conducted a nationwide study of mHealth apps in China. The aim of this study was (1) to describe and compare digital health concerns before and after the COVID-19 outbreak, including mHealth app data flow and user experience, and (2) to analyze the impact of COVID-19 on mHealth apps.

## Methods

### Data Acquisition

Before the COVID-19 outbreak, we conducted a comprehensive electronic search of 4 sources up to October 25, 2019: (1) apps on leaderboards of health-related categories in the 6 largest app markets in China, including the top 50 on the Huawei Android Market (Huawei Technologies Co Ltd; Shenzhen, China), top 50 on the OPPO Android Market (Oppo Electronics Co Ltd; Dongguan, Guangdong, China), top 100 on the Vivo Android Market (Vivo Co Ltd; Dongguan, Guangdong, China), top 50 on the Tencent Android Market (Tencent Holdings Limited; Shenzhen, China), top 100 on the 360 Android Market (Qihoo 360 Technology Co Ltd; Beijing, China), and top 100 on the Apple iTunes store for China (Apple; Cupertino, CA) [23]; (2) the mHealth apps on the first 20 web pages of the Baidu search engine, which is the largest search engine in China; (3) apps that can connect to smart devices from the 4 large online shopping sites (Tmall, JD, Pinduoduo, and Suning); and (4) apps affiliated with internet hospitals on the list of the top 100 Chinese hospitals in 2018. The exclusion criteria were (1) duplicated apps, (2) app descriptions irrelevant to health, (3) apps not available in the Chinese language, (4) apps that were not available for download through the official Android and Apple app stores or the Baidu search engine, and (5) apps that could not be opened or used due to technical problems. Apps with patient and clinician versions were evaluated as different items. Search terms, sample quantities, and data collection times for each source are provided in Table S1 in Multimedia Appendix 1.

We collected 4 types of data: (1) basic app characteristics from the description interface, including the size of apps, number of app downloads, and target users; (2) app developers’ information from the largest commercial inquiry platform, the Tianyancha website [24], including transaction amount, registered capital, number of staff, operating status, establishment date, and geographic location; (3) app permission listing data from the permission interface; and (4) app functions from app trials, except for apps only open to internal users. As the iOS App Store does not display the number of downloads, we replaced total downloads in iOS with the number of reviews and used statements of “downloads” in the following paragraphs. If an app existed on multiple platforms, the total number of app downloads was calculated as the sum of app downloads from all platforms. The app trial included at least two investigators who downloaded selected applications and independently identified application functionality according to a clear functional definition by using iPhones and Android phones. See Table S2 in Multimedia Appendix 1 for details on function definitions.

Following the outbreak, the same mHealth apps were investigated a second time in April 2021 as semilongitudinal samples with download data, and we determined whether COVID-19 content had been added. A download change was defined as the difference between post and prepandemic downloads.

### User Experience Survey

We recruited residents through a community health checkup program to explore the user experience with mHealth apps among a large community of more than 20,000 people with a balanced age distribution in southeastern China. We used an offline questionnaire to survey 400 participants from October 2019 to November 2019 before the COVID-19 outbreak and 200 participants from June 2020 to August 2020 after the outbreak. A total of 553 (553/600, 92.2%) participants completed the survey: 375 (375/400, 93.8%) before the outbreak and 178 (178/200, 89.0%) after the outbreak. A predesigned, structured questionnaire was provided to potential participants in the waiting areas of the medical examination center in this community. The questionnaire was designed to collect information on participants’ attention to health information, various aspects of mHealth technology usage, willingness to consume mHealth technology, and health status and demographic characteristics. Trained research assistants who were fluent in Chinese administered the questionnaire and provided verbal instructions about how to complete them.

### Ethical Considerations

Before taking the survey, informed consent was obtained from each participant. This study was approved by the Biomedical Research Ethics Committee of Fujian Medical University (2018 number 11). All procedures were performed using the relevant guidelines and regulations.

### Data Analysis

A descriptive analysis was conducted for mHealth app characteristics, developers, permission, functions, and user experiences in China. Data are presented using frequencies and percentages, bar charts, statistical maps, Venn diagrams, and heat maps. Continuous variables are presented as the mean and SD or the median and IQR, while categorical variables are presented as the frequency and percentage. Mann-Whitney *U* tests or chi-square tests were used to assess differences among variables.

We also compared the post and prepandemic app downloads of each category using paired *t* tests. To account for multiple comparisons, we calculated a Bonferroni-corrected *P* value criterion of .05/28. Therefore, *P*<1.79x10^-3^ was considered statistically significant.

Quantile regression (QR) models were used to explore the relationship between modeling covariates and quartiles of the outcome variable of interest [25]. Because of the wide range and non-normal distribution of download changes, we used QR models to examine associations between app characteristics, functions, and outcomes at specific quantiles of distribution in download changes. This analysis does not make assumptions about the residual distribution and is more robust to outliers in the outcome [26]. Unlike an ordinary linear regression model, which models the mean of only 1 dependent variable, QR examines the effect of covariates at different points of the conditional distribution of the response variable and gives more comprehensive results. QR models provided a more detailed view of associations of app characteristics, functions, and outcomes with download changes. We obtained estimates and plotted them at the following quantiles: 10th, 25th, 50th (median), 75th, and 90th. In the QR analysis, significance was assessed at the 10% level.

All analyses were prespecified and performed using SPSS 25.0 (IBM Corp; Armonk, NY) and Stata version 13 (StataCorp; College Station, TX).

## Results

### App Characteristics

**Figure 1:**
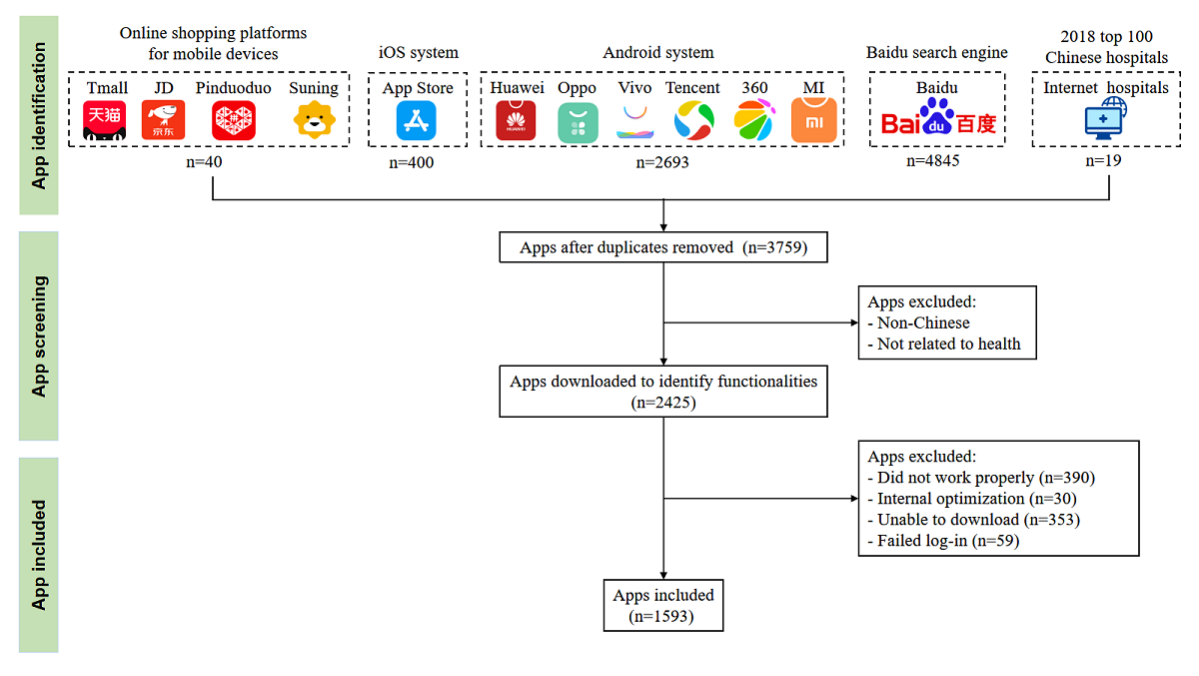
Flow diagram of app selection.

**Figure 2:**
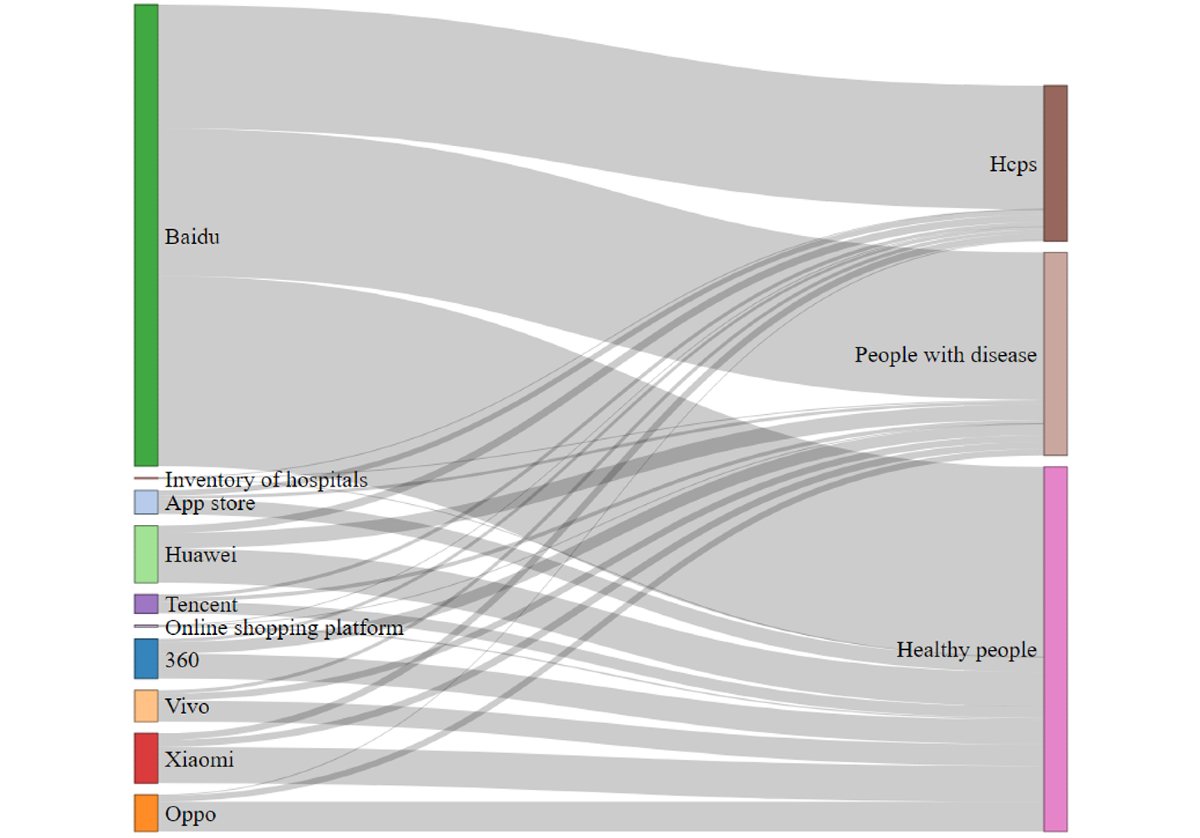
Sankey diagram of flow direction in apps. The width of the colored boxes and their connecting gray bands are directly proportional to the frequency of apps from every data source (left side) and flow quantities of these apps to the attributable user communities (right side). Hcps: health care professionals.

**Figure 3:**
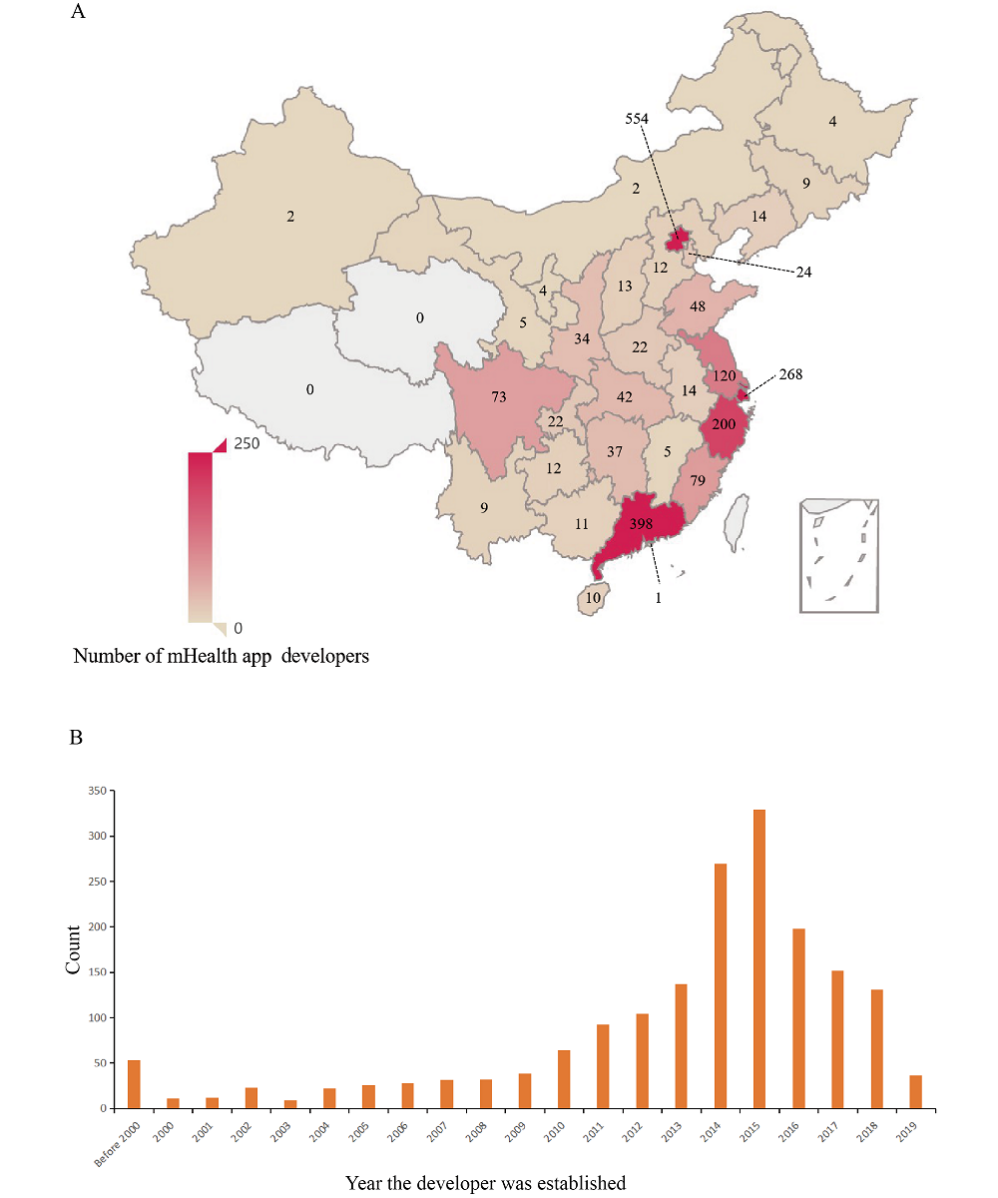
(A) Geographical distribution and (B) distribution of the time of establishment of mobile health (mHealth) app developers in China. There is another developer in Canada.

A total of 1593 mHealth apps were included in the analysis (see Figure 1). During the COVID-19 pandemic, various app downloads showed an overall upward trend (change in median: 61,561), and approximately 10% (182/1593, 11.4%) of the apps had functions or content added for COVID-19 (see Table 1). Figures 2 and 3 show the target users and geographic distribution of the apps. Target users included healthy people (921/1593, 57.8%), patients (513/1593, 32.2%), and health care professionals (393/1593, 24.7%). The geographical distribution of mHealth app developers included in the study was concentrated in megacities (ie, Beijing, Shanghai, Guangzhou) and southeast China (coastal areas with a developed economy; see Figure 3). Additional permission requests were found in app markets (see Table S3 in Multimedia Appendix 1).

**Table 1:** Characteristics and developers of mobile health (mHealth) apps available on the Chinese market (1593 apps and 1196 developers).

Characteristics			Results
**Basic information**			
	Size of app (MB), mean (SD)		37.26 (75.46)
	Size of app (MB), median (IQR)		24.90 (13.50-39.88)
	**Download price, n (%)**		
		Free download	1530 (96.0)
		Paid	63 (4.0)
	If download is free, in-app purchase available, n (%)		1444 (90.6)
	In-app advertisement, n (%)		175 (11.0)
	**Target users, n (%)**		
		Medical researchers	72 (4.5)
		Medical personnel	100 (6.3)
		Patients	513 (32.2)
		Healthy people	921 (57.8)
	Has a membership system, n (%)		101 (6.3)
	Connects to smart devices, n (%)		292 (18.3)
	User rating score, mean (SD)		6.99 (2.33)
	User rating score, median (IQR)		7.00 (6.00-9.20)
	**Total downloads (x10^3^)**		
		Prepandemic, mean (SD)	8967.33 (118,905.97)
		Prepandemic, median (IQR)	44.09 (1.47-787.00)
		Postpandemic, mean (SD)	17,342.13 (275,177.12)
		Postpandemic, median (IQR)	223.12 (10.93-2252.00)
	Number of functions, mean (SD)		2.72 (2.25)
	Number of functions, median (IQR)		2 (1-4)
	Added functions or content for the pandemic, n (%)		182 (11.4)
	**Type of function or content added for the pandemic, n (%)**		
		Pandemic consultation	105 (6.6)
		Pandemic prevention knowledge	108 (6.8)
		Pandemic dynamics	9 (0.6)
**App developers**			
	Transaction amount (¥; x10^6^)^a^, mean (SD)		267.66 (812.75)
	Transaction amount (¥; x10^6^)^a^, median (IQR)		50.00 (20.50-150.00)
	Registered capital (¥; x10^6^)^a^, mean (SD)		188.10 (204.15)
	Registered capital (¥; x10^6^)^a^, median (IQR)		937.50 (166.00-210.53)
	Number of staff, mean (SD)		262 (3016)
	Number of staff, median (IQR)		17 (2-62)
	**Operating status, n (%)**		
		In business	482 (30.3)
		Remainder enterprise	697 (43.8)
		Closed down	15 (1.0)

^a^A currency exchange rate of ¥1=US $0.15 is applicable.

**Figure 4:**
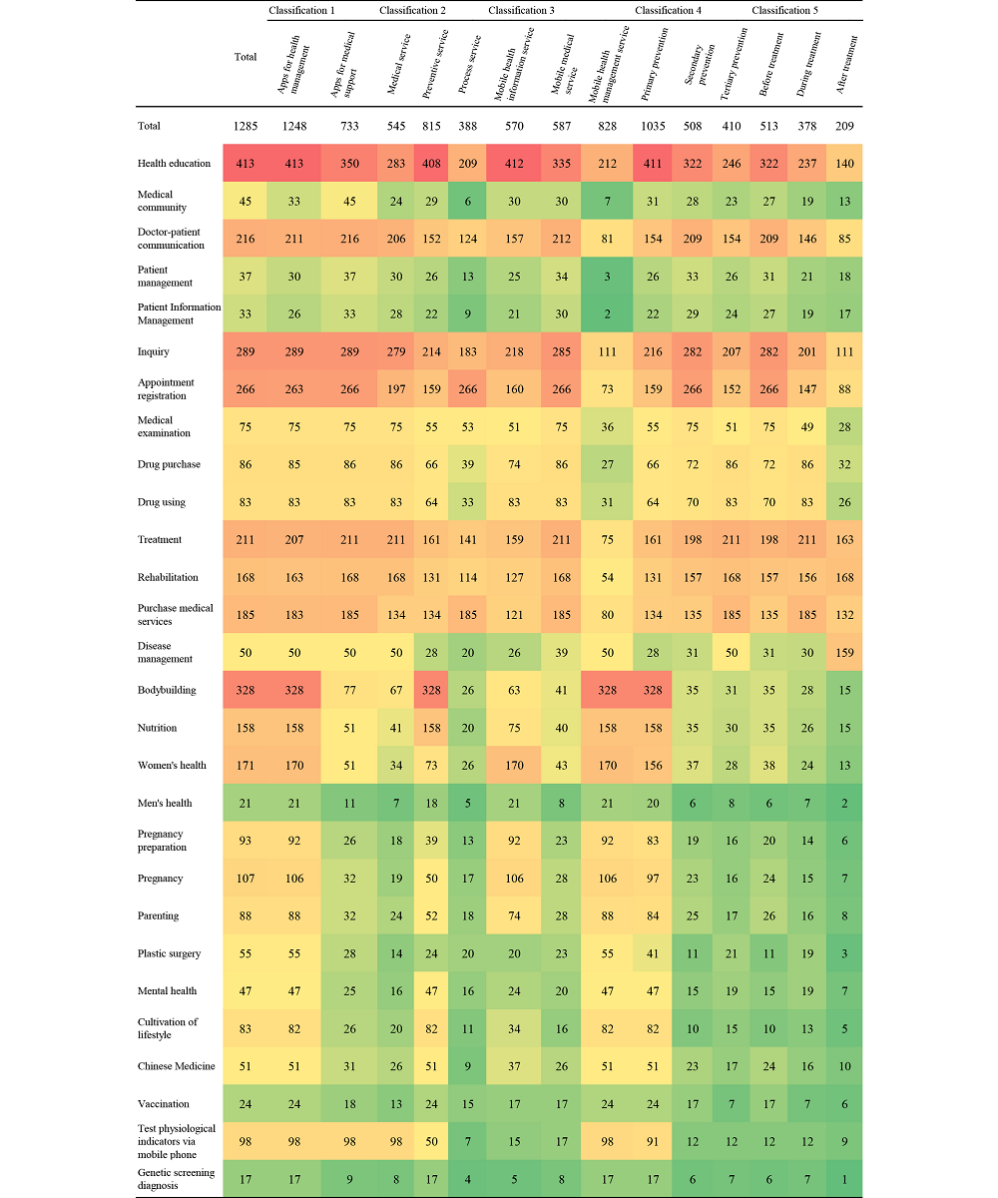
For each classification of mobile health (mHealth) apps in China: (A) frequency of app functions for 5 classifications; (B) ranking of the frequency of app functions is displayed on a color scale ranging from green (lowest charge rates) to orange, yellow, and red (highest charge rates).

**Figure 5:**
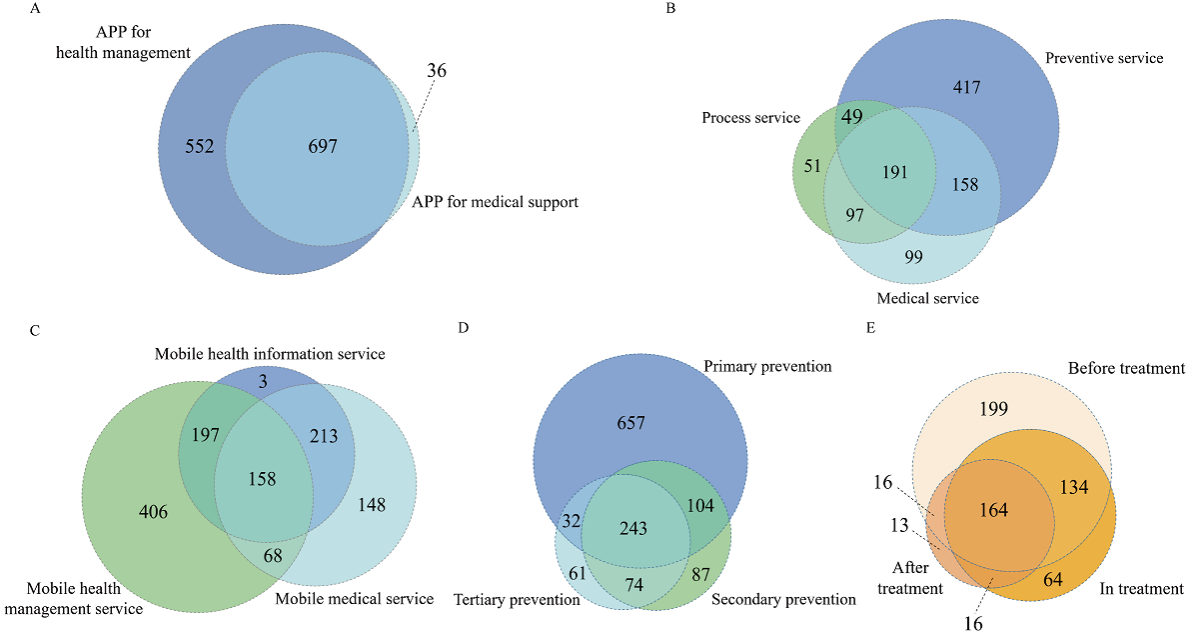
Venn diagrams illustrating the associations between app classifications: (A) user communities, (B) mobile health service function, (C) content or services provided by apps, (D) tertiary prevention, and (E) service time provided by apps.

Of the 1593 mHealth apps, 1285 (80.7%) apps had full functionality available to conduct the app trial, including apps for health management (1248/1593, 78.3%) and apps for medical support (697/1593, 43.8%). The frequency of each function available in Chinese mHealth apps is shown in Figure 4. Figure 5 shows the associations between the 5 app classifications, with the circles scaled to the number of apps in each app classification. The rating concepts for these 5 different classifications in app trials are available in Table S4 in Multimedia Appendix 1 [27].

### Comparison of Download Changes in Apps With Different Functions Between Pre and Postpandemic

**Figure 6:**
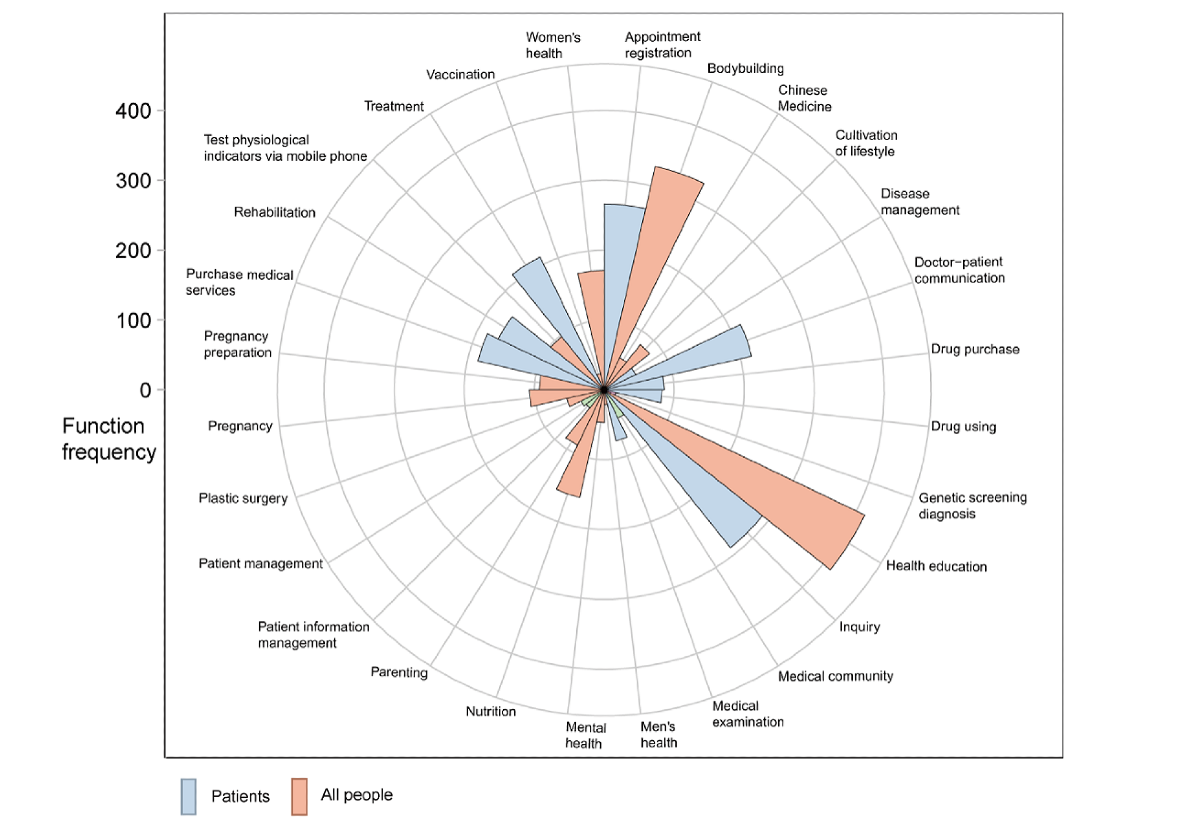
The number of occurrences of each function (ie, function frequency) in the 1593 mobile health (mHealth) apps with multiple functions in China.

**Figure 7:**
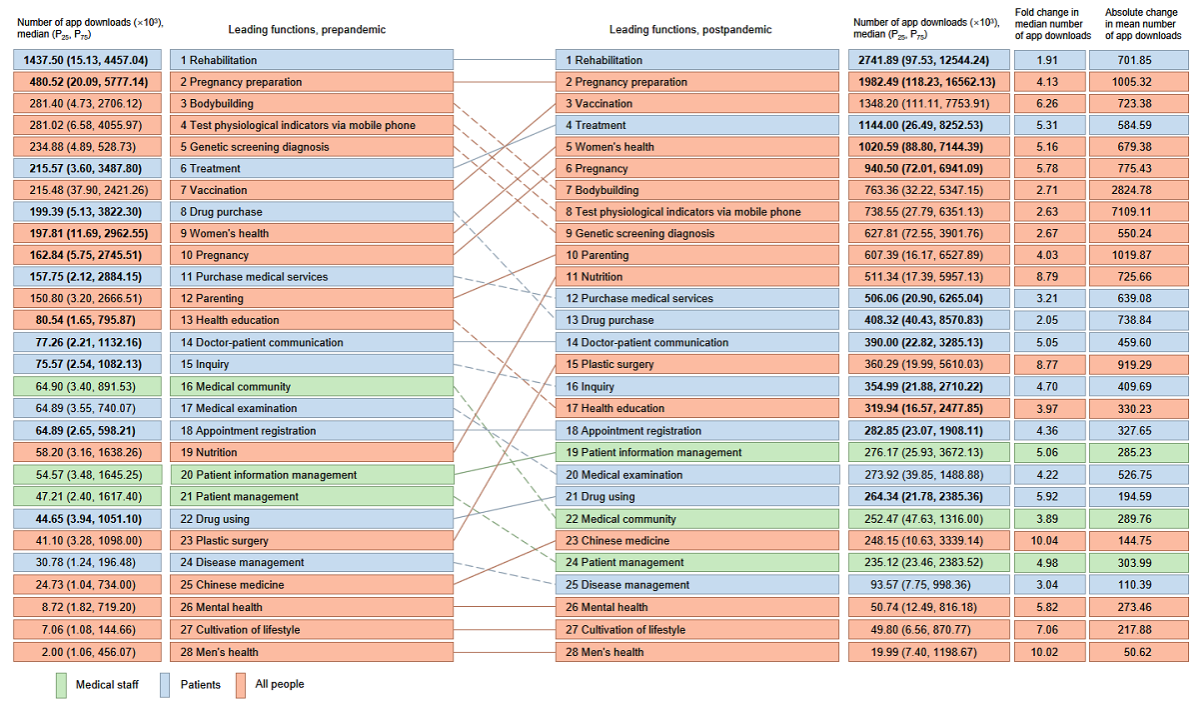
Leading functions of mobile health (mHealth) apps in the pre and postpandemic periods in China, connected by lines between the periods to show increased (solid line) or decreased (dashed line) ranking, while bold number indicate significant changes between the periods as determined using paired *t* tests with Bonferroni-corrected *P* values <1.79x10^-3^. Fold change in median of the number of app downloads=(Medpost-Medpre)/Medpre. Absolute change in mean of the number of app downloads=∑(npost-npre)/n.

Overall upward trends in app downloads during the pandemic were driven by some key app functions (see Figures 6 and 7). Rehabilitation (prepandemic median 1,437,500; postpandemic median 2,741,890; *P*<.001) and pregnancy preparation (prepandemic median 480,520; postpandemic median 1,982,490; *P*<.001) were the most often used functions, with statistically significant differences between pre and postpandemic. The 9 most important drivers for increasing downloads were divided into 2 categories. Three functions related to children and maternal health were observed: women's health (fold change 5.16), pregnancy (fold change 5.78), and parenting (fold change 4.03). Increased downloads of these apps (women’s health, *P*<.001; pregnancy, *P*<.001; parenting, *P*<.001) were also observed between pre and postpandemic. The other 6 most popular functions regarding treatment needs and maintenance were treatment (fold change 5.31), plastic surgery (fold change 8.77), drug use (fold change 5.92), patient information management (fold change 5.06), nutrition (fold change 8.79), and Chinese medicine (fold change 10.04). Vaccination, which was most relevant to COVID-19, saw a large spike in download rates, with an increase of 6.26 times (absolute change in mean: 7,233,800) postpandemic compared with prepandemic.

The 6 functions that decreased in ranking the most in the postpandemic period were health education, genetic screening, medical service purchases, drug purchases, inquiries, and physiological testing via mobile phone; 4 additional functions that declined after the COVID-19 outbreak included medical examination, medical community, patient management, and disease management. Bodybuilding, which was closely related to outdoor activities, also declined in ranking, with an increase of only 2.71 times prepandemic rates, but its absolute change in mean was 28,247,800. All these functions with declining rankings were growing, albeit at lower speeds relative to high-ranking functions. For example, the increase in drug purchases was 2.05 times (absolute change in mean: 7,388,400) the prepandemic rate, and patient management downloads were 4.98 times (absolute change in mean: 3,039,900) the prepandemic rate.

### Relationship Between App Characteristics and Download Changes

**Figure 8:**
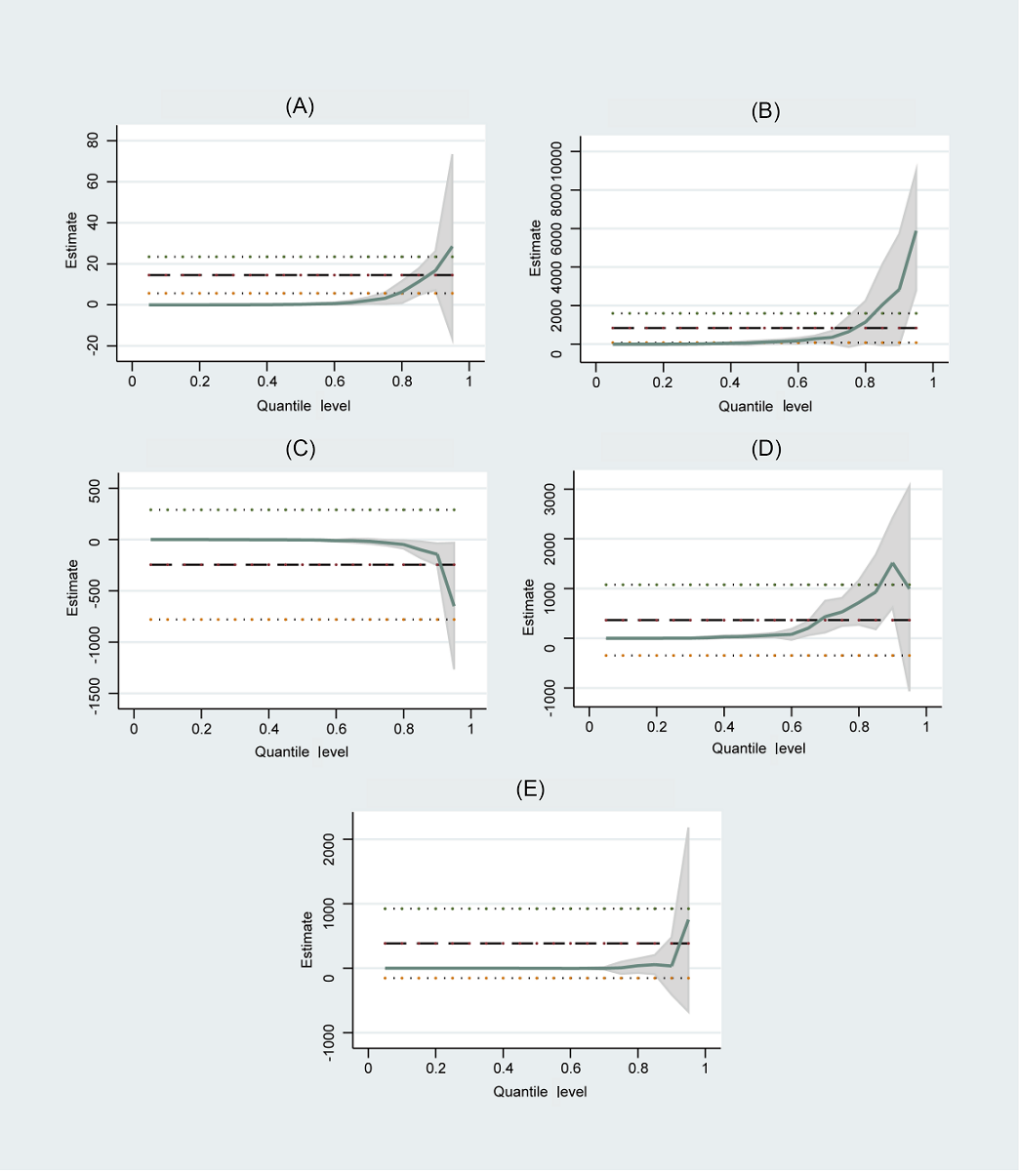
Statistically significant differential effects of app characteristics by quantile: (A) app size, (B) in-app advertisement, (C) number of functions, (D) functions or content added for the pandemic, (E) Internet of Things.

Based on the QR analysis, a positive effect for adding COVID-19 function and content on apps (P_10_: *P*=.001; P_50_: *P*=.01; P_75_: *P*=.009; P_90_: *P*=.01) was observed across download changes in most quantiles, with the largest effect at the 90th quantile (see Table 2, Figure 8, and Multimedia Appendix 2). Positive effects of the size of apps on download changes were also observed at high quantiles (P_75_: *P*=.03; P_90_: *P*=.002). Other characteristics of the QR are shown in Table 2.

**Table 2:** Quantile regression coefficients of app characteristics for changes in downloads.

App characteristics		Quantiles											
		0.10		0.25		0.50		0.75		0.90			
**Operating status**													
	Coefficient (x10^4^; 95% CI)		0.000142 (–0.151 to 0.151)		–0.0428 (–0.500 to 0.414)		0.809 (–2.786 to 4.404)		–4.951 (–28.311 to 18.409)		–20.304 (–120.276 to 79.669)		
	*P* value^a^		>.99		.85		.66		.68		.69		
**Size of app**													
	Coefficient (x10^4^; 95% CI)		0.0000367 (–0.005 to 0.005)		0.0102 (–0.018 to 0.038)		0.256 (–0.041 to 0.554)		3.173 (0.239 to 6.108)		16.728 (6.124 to 27.332)		
	*P* value^a^		.99		.45		.09		.03		.002		
**In-app advertisement**													
	Coefficient (x10^4^; 95% CI)		–0.0857 (–0.321 to 0.150)		1.072 (–11.297 to 13.441)		101.809 (15.949 to 187.668)		648.489 (–267.967 to 1564.944)		2840.221 (–213.048 to 5898.490)		
	*P* value^a^		.48		.87		.02		.17		.07		
**Number of functions**													
	Coefficient (x10^4^; 95% CI)		–0.0504 (–0.252 to 0.151)		–0.691 (–1.340 to –0.041)		–3.501 (–9.198 to 2.196)		–31.571 (–69.032 to 5.889)		–143.767 (–321.698 to 34.165)		
	*P* value^a^		.62		.04		.23		.098		.11		
**Prevention classification**													
	Coefficient (x10^4^; 95% CI)		0.0258 (–0.019 to 0.071)		0.0593 (–0.147 to 0.265)		1.675 (–0.448 to 3.798)		7.928 (–10.998 to 26.855)		66.478 (–22.249 to 155.205)		
	*P* value^a^		.26		.57		.12		.41		.14		
**Connect to smart devices**													
	Coefficient (x10^4^; 95% CI)		0.337 (0.008 to 0.665)		0.166 (–0.323 to 0.655)		–1.576 (–6.213 to 3.062)		5.069 (–51.167 to 61.305)		34.596 (–256.520 to 325.712)		
	*P* value^a^		.045		.51		.51		.86		.82		
**Added functions or contents for pandemic**													
	Coefficient (x10^4^; 95% CI)		0.450 (0.190 to 0.710)		1.788 (–3.121 to 6.698)		46.627 (9.800 to 83.453)		529.365 (132.535 to 926.194)		1517.683 (352.991 to 2682.376)		
	*P* value^a^		.001		.48		.01		.009		.01		

^a^Significance was assessed at the 10% level.

### Relationship Between App Functions and Download Changes

**Figure 9:**
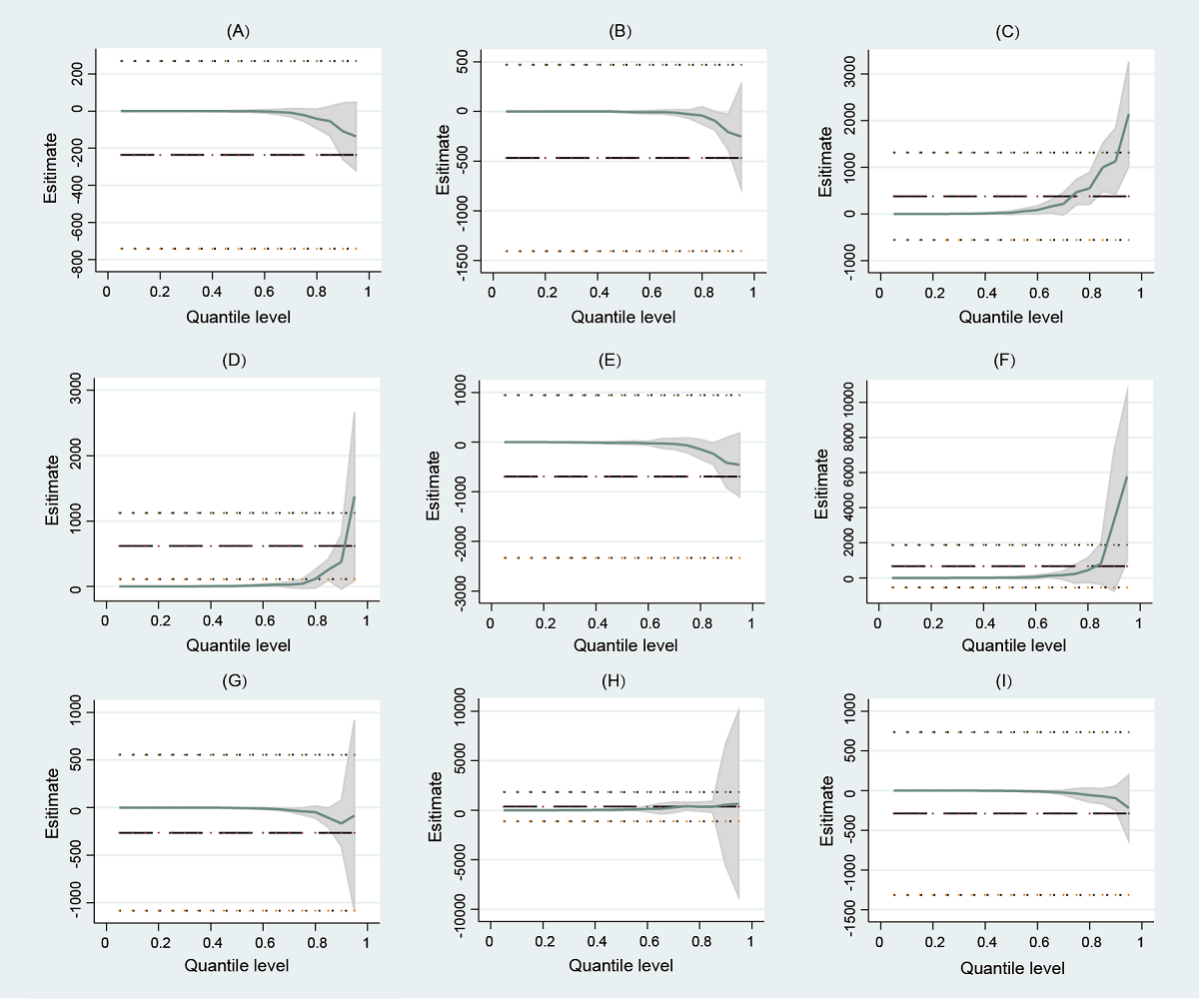
Statistically significant differential effects of app functions by quantile: (A) health education, (B) drug use, (C) rehabilitation, (D) bodybuilding, (E) men’s health, (F) pregnancy preparation, (G) cultivation of lifestyle, (H) vaccination, (I) disease management.

We found that 4 of 28 functions, including rehabilitation (P_75_: *P*=.003; P_90_: *P*=.02), pregnancy preparation (P_90_: *P*=.09), bodybuilding (P_50_: *P*=.07; P_90_: *P*=.08), and vaccination (P_75_: *P*=.06), were positively associated with download changes, mainly in the higher quantiles (see Table 3, Figure 9, and Multimedia Appendix 3). Health education (P_75_: *P*=.09; P_90_: *P*=.09), drug use (P_90_: *P*=.08), cultivation of lifestyle (P_25_: *P*=.02; P_50_: *P*=.06; P_75_: *P*=.07), men’s health (P_90_: *P*=.07), and disease management (P_75_: *P*=.03) had negative correlations with download changes. Other functions in the QR are shown in Table S5 in Multimedia Appendix 1.

**Table 3:** Quantile regression coefficients of app functions for changes in downloads.

App functions		Quantiles				
		0.10	0.25	0.50	0.75	0.90
**Health education**						
	Coefficient (x10^4^; 95% CI)	0.0002 (–0.0687 to 0.0691)	0.0292 (–0.308 to 0.367)	–1.036 (–4.364 to 2.293)	–21.668 (–46.305 to 2.969)	–108.990 (–234.145 to 16.166)
	*P* value^a^	>.99	.87	.54	.09	.09
**Drug use**						
	Coefficient (x10^4^; 95% CI)	0.0739 (–0.388 to 0.536)	0.133 (–0.866 to 1.132)	–3.450 (–11.716 to 4.816)	–26.942 (–103.928 to 50.045)	–207.444 (–436.652 to 21.764)
	*P* value^a^	.75	.79	.41	.49	.08
**Rehabilitation**						
	Coefficient (x10^4^; 95% CI)	0.0992 (–0.218 to 0.417)	1.624 (–0.807 to 4.055)	26.345 (–10.549 to 63.239)	470.025 (160.786 to 779.265)	1132.3840 (172.086 to 2092.682)
	*P* value^a^	.54	.19	.16	.003	.02
**Bodybuilding**						
	Coefficient (x10^4^; 95% CI)	0.0004 (–0.0324 to 0.0332)	0.0219 (–0.375 to 0.419)	7.194 (–0.472 to 14.861)	41.692 (–39.050 to 122.433)	376.090 (–49.408 to 801.587)
	*P* value^a^	.98	.91	.07	.31	.08
**Men's health**						
	Coefficient (x10^4^; 95% CI)	–0.0047 (–0.0324 to 0.0332)	–1.497 (–5.075 to 2.081)	–11.928 (–45.420 to 21.565)	–65.017 (–242.440 to 112.407)	–415.092 (–869.710 to 39.527)
	*P* value^a^	>.99	.41	.49	.47	.07
**Pregnancy preparation**						
	Coefficient (x10^4^; 95% CI)	0.1684 (–1.938 to 2.275)	3.037 (–1.692 to 7.765)	16.135 (–26.886 to 59.157)	226.703 (–248.826 to 702.233)	3257.672 (–446.489 to 6961.834)
	*P* value^a^	.88	.21	.46	.35	.09
**Cultivation of lifestyle**						
	Coefficient (x10^4^; 95% CI)	8.35^b^ (–0.0418 to 0.0418)	–0.445 (–0.803 to –0.0872)	–4.078 (–8.238 to 0.083)	–39.345 (–81.525 to 2.834)	–166.124 (–461.988 to 129.740)
	*P* value^a^	>.99	.02	.06	.07	.27
**Vaccination**						
	Coefficient (x10^4^; 95% CI)	0.0046 (–5.995 to 6.004)	7.758 (–18.734 to 34.251)	44.707 (–139.634 to 229.048)	410.179 (–18.195 to 838.553)	566.790 (–5041.161 to 6174.740)
	*P* value^a^	>.99	.57	.63	.06	.84
**Disease management**						
	Coefficient (x10^4^; 95% CI)	0.0418 (–0.377 to 0.460)	–0.102 (–0.856 to 0.652)	–4.011 (–9.643 to 1.622)	–37.508 (–71.617 to –3.398)	–95.521 (–237.203 to 46.161)
	*P* value^a^	.85	.79	.16	.03	.19

^a^Significance was assessed at the 10% level.

^b^10^-17^.

### User Experience

No significant difference was found in the sex (*P*=.41) or mean age (*P*=.52) of the participants. The attention given to health information (prepandemic: 249/375, 66.4%; postpandemic: 146/178, 82.0%; *P*=.006) and the percentage of people owning smartphones (prepandemic: 186/375, 49.7%; postpandemic: 108/178, 60.7%; *P*=.02) steadily increased after the outbreak (see Table 4). The vast majority of individuals (prepandemic: 129/141, 92.1%; postpandemic: 89/90, 98.9%) used social networks (eg, WeChat) to obtain health information online. Furthermore, the ratio of internet hospital use rose dramatically (prepandemic: 6/375, 1.6%; post-pandemic: 23/178, 12.9%; *P*<.001). Other characteristics of the user experience analysis are shown in Table 4.

**Table 4:** Population use of mobile health (mHealth) apps in the pre and postpandemic periods.

Characteristics			Before the outbreak (n=375)		After the outbreak (n=178)		*P* value
Age (years), median (IQR)			70 (66-74)		70 (66-75)		.52
Age (years), mean (SD)			70.46 (6.329)		70.85 (7.680)		—^a^
**Sex, n (%)**							.41
	Male	166 (44.3)		85 (48.0)			
	Female	209 (55.7)		92 (52.0)			
**Attention to health information, n (%)**							.006
	Never	125 (33.3)		32 (18.0)			
	Sometimes	131 (34.9)		75 (42.1)			
	Often	73 (19.5)		57 (32.0)			
	Always	45 (12.0)		14 ( 7.9)			
The ability to use electronic products (mobile phone, tablet, computer), n (%)			186 (49.7)		108 (60.7)		.02
Getting health information offline in the past 6 months, n (%)			108 (28.8)		104 (58.4)		<.001
Getting health information through the internet in the past 6 months, n (%)			141 (37.6)		90 (50.6)		.004
**Ways to obtain health information, n (%)**							
	Social network (eg, WeChat, QQ)	129 (92.1)		89 (98.8)		.008	
	Portal web	36 (25.9)		19 (22.6)		.63	
	mHealth apps	6 ( 4.3)		2 ( 2.5)		.71	
	Search engine	36 (25.5)		22 (26.5)		.87	
	Internet hospitals	6 ( 1.6)		23 (12.9)		<.001	
**Consuming intention for internet health information per month (¥^b^), n (%)**							.70
	0	316 (84.3)		150 (84.3)			
	1-10	10 (2.7)		5 (2.8)			
	11-50	19 (5.1)		9 (5.1)			
	51-100	3 (0.8)		0 (0)			
	101-200	2 (0.5)		1 (0.6)			
	201-500	1 (0.3)		0 (0)			
	>500	0 (0)		0 (0)			
**The actual cost for internet health information last month (¥^b^), n (%)**							.45
	0	348 (92.8)		164 (92.1)			
	1-10	0 (0)		2 (1.1)			
	11-50	0 (0)		2 (1.1)			
	51-100	1 (0.3)		0 (0)			
	101-200	1 (0.3)		0 (0)			
	201-500	1 (0.3)		0 (0)			
	>500	1 (0.3)		0 (0)			
**Intention to avoid some unhealthy behaviors after** **obtaining health information from the internet, n (%)**							.67
	Never	15 (4.0)		0 (0)			
	Sometimes	12 (3.2)		16 (9.0)			
	Often	65 (17.3)		47 (26.4)			
	Always	25 (6.7)		9 (5.0)			

^a^Age was not normally distributed, so differences in the median value were assessed using a nonparametric test.

^b^A currency exchange rate of ¥1=US $0.15 is applicable.

## Discussion

### Principal Findings

Our study demonstrates that the usage and population utilization of mHealth applications increased after the COVID-19 outbreak. As a powerful tool for providing health care services, functions closely related to the pandemic, including rehabilitation, treatment, drug use, and vaccination, were positively associated with changes in app downloads. The high growth of app use related to maternal and child health, including pregnancy preparation and women’s health, shows the potentially increased desire for family among the Chinese population in the postpandemic era. Moreover, the user experience and high use of health management apps also reflect great attention to self-care. Overall, mHealth apps assist with health improvement against the background of normalized pandemic control and may improve fertility.

### COVID-19–Related Apps

The usage of COVID-19 pandemic-related apps, such as vaccination, increased in rank. Furthermore, adding pandemic-related functions positively correlated with increased downloads. The likely reason behind this rise was that apps inherently related to the pandemic can easily capture the attention of the public as a means of obtaining information. Some apps with larger user groups may also add COVID-19 modules to respond to normalized pandemic prevention and control policies [14].

### Medical Support Apps

Unprecedented large-scale quarantine measures and shortages of medical resources have made telemedicine care an important and real demand during the pandemic [28]. In our research, rehabilitation, including apps for long-term care or chronic disease management, was the most widely used function before and after the outbreak, overcoming the interruption of personal health care services caused by COVID-19. mHealth apps are used for many rehabilitation purposes [25]**.** One study showed that effective rehabilitation apps helped patients increase their health and happiness index during the pandemic period [29]. Furthermore, the use of mHealth apps can help improve adherence to treatment [30]. Use of apps with functions for rehabilitation, treatment, and drug use increased significantly after the outbreak, providing stable medical services to reduce the negative impact of home isolation. China’s emerging internet model provides the basis for remote pharmacy services [31], assisting patients who cannot always go to the pharmacy. At the same time, personalized medical plans are also an important part of precision medicine [32]. The city of Taizhou, China, had a successful experience using telemedicine to prevent and treat COVID-19 [33]. Consequently, mHealth apps are an effective medical tool in the context of COVID-19.

### Health Management Apps

Most apps were designed for health management by all people, mainly focusing on bodybuilding and nutrition. In our research, app flow and user experience surveys both showed that the rankings of most functions related to health management were rising, which reflects great attention to self-care postpandemic.

It is interesting that there was increased use of maternal and child health apps, including pregnancy preparation, women’s health, pregnancy, and parenting, after the outbreak, showing potentially increased desire for fertility among the Chinese population.

One study concluded that the pandemic is affecting people’s desire to become parents, which was consistent with our results [34]. Another previous study conducted a cross-sectional survey of 285 apps to analyze the current situation of maternity apps in Italy, finding that high-quality, targeted, and effective apps for pregnancy and postnatal health care had relevant implications in terms of maternal and newborn health prevention and promotion [35]. mHealth apps have the potential to be used extensively in improving maternal well-being [36]. The high growth of maternal and child health-related apps such as pregnancy preparation and women's health in our study coincides with this. Before the pandemic, the progression of population aging, decline of women in the childbearing period, and increase in high-risk pregnancies had led to the continuous decline in China’s fertility rate [21]. Women have suffered significant reproductive health disruption since the beginning of the COVID-19 pandemic [37,38], which may have aggravated the decline in fertility [39]. However, with long-term home isolation and 2-child and 3-child policies proposed by the government, people are giving more attention to family matters, including pregnancy preparation and raising children, which may slow the decline in fertility to a certain extent. Therefore, maternal and child health apps can effectively assist health management and fill the vacancy of in-person perinatal health care services [40], despite interrupted pregnancy checkups [39]. It has been suggested that a model of health management combined with continuous care using the WeChat platform can significantly improve patients’ postoperative medication compliance and quality of life by requiring them to complete their rehabilitation tasks in the WeChat group, which is worth applying and promoting [41]. In China, pregnant women are accustomed to using WeChat groups recommended by hospitals to discuss pregnancy health information and may promote apps in it, which may be a way to increase app usage.

For bodybuilding apps, this function had positive changes in downloads during the COVID-19 pandemic. One study found that the keyword “mHealth” was closely associated with “physical activity” and “ehealth” in the last 2 decades of research on digital health behavior change technologies [42]. Closed gyms and restricted outdoor activities during the pandemic reduced physical activity levels; however, the use of physical activity apps may counteract the decline in exercise [43].

Many kinds of apps attempted to provide health education, which was the most widely available function in our study. Although this function declined during the pandemic and had negative changes in downloads, the population’s attention to health information increased. The probable cause behind this phenomenon is that new media platforms in China, such as WeChat and Weibo, have been vigorously promoted as important means for pandemic-related health information dissemination, which may decrease interest in acquiring apps when the information is readily available on these platforms. The high levels of knowledge of the Chinese public about COVID-19 prevention mainly comes from WeChat [44]. This is consistent with the results of our study that people use WeChat most often to obtain health information.

The COVID-19 pandemic has caused health anxiety at the population level. Digital intervention by mHealth apps is suitable for alleviating such sociopsychological consequences [45]. However, it may be because of the vigorous development of the psychological counseling hotline project in China during the pandemic that mental health apps were not of great concern. Furthermore, a study that conducted a systematic assessment of self-guided cognitive behavioral therapy (CBT)–based apps concluded that only a few self-guided CBT-based apps offer comprehensive CBT programs or suicide risk management resources, which may also be one of the reasons for not getting much attention [46].

### Willingness to Use mHealth Apps

Consistent with the report of the rapid increase in older adult internet users during the COVID-19 pandemic in China, we found that people over 50 years old paid more attention to mHealth apps after the outbreak [47-50]. In conjunction with the increased interest of older adults in the mHealth space, the results of a previous study suggested that person-centered mHealth apps can be used to create mHealth solutions with positive outcomes for older adults [51]**.** Coupled with the higher probability of chronic diseases or other conditions, older individuals should be a primary and adaptable group for mHealth apps [52]. However, the digital divide makes optimizing mHealth apps among older individuals difficult [53]. The Chinese people strongly support public health measures proposed due to COVID-19, which makes it possible to develop the ability to use smart devices, reduce the digital divide, and provide QR codes for pass certificates [54]. It is worth noting that a larger software file size was positively associated with download changes, indicating that excessive software size has little effect on the willingness to use the software. Having the right function is what users are most concerned about, and the causal relationship needs to be further studied [55].

### Limitations

Our study should be considered in the context of important limitations. First, this study excluded apps that were used internally by medical staff because we could not log in as an internal account holder; this dilutes the results of mHealth apps designed for health care professionals. However, our research focused on apps for patients and healthy people rather than internal apps. Future work will be conducted with apps used internally in medical care. Second, the absence of newly emerging apps made it impossible to provide an overview of the mHealth market after the COVID-19 outbreak. Therefore, we compared the changes in downloads during the pre and postpandemic periods to explore the relationship between various types of apps and the pandemic. Third, as a semilongitudinal survey, this study measured exposure and outcome, and it was difficult to derive causal relationships from the analysis; thus, we only made assumptions based on the status quo.

### Strengths

The study has practical implications and applications. This study is the first to investigate the relationship between COVID-19 and population-level utilization of mHealth apps through a semilongitudinal study of app markets’ data and a field questionnaire, combined with the results of both the web-based survey and the population user experience survey. As China is one of the few countries to adopt more active public health prevention and control measures, this study, which involves a multilevel and broad research scope, can provide strong data support for future comparative studies between different countries and regions. In the user experience survey, we explored the changing attitudes of the population toward digital health technology, suggesting that there is a good development environment for mHealth apps in the postpandemic era. This study, with consistent definitions of variables and processes, allowed the investigators to consistently classify mHealth apps and ensure data integrity, underpinning its strength. Our research clarified the relationship between various types of apps and usage changes by conducting investigations in the pre and postpandemic periods. We believe our results provide a good reference for the subsequent development of future mHealth apps. In addition to the increasing number of COVID-19–related apps prompted by pandemic policies, app developers should be aware that maternal, child, and self-care management are app functions about which the population is concerned.

### Perspective

mHealth apps utilize information and telecommunications technology to transfer medical information for diagnosis, therapy, and education and played a significant role following the COVID-19 outbreak. The pandemic made people aware of the value of mHealth in promoting universal health coverage, which promotes stronger management of self-care. Against the backdrop of an increased desire to raise a family among the Chinese population in the postpandemic era, maternal and child health apps, as a health education tool, promote a healthy lifestyle for women’s self-management in the antenatal and postpartum periods. Further research is needed to understand the users’ requirements for these apps, which will influence their adoption. The explicit design of apps is another potential factor that can facilitate or hinder user engagement and requires further investigation.

### Conclusion

mHealth apps are an effective approach to providing health care in the context of COVID-19. This study clarifies the increasing usage of different apps during the pre and postpandemic periods, showing greater attention to self-care and the Chinese population’s increasing desire to raise a family. Moreover, our research provides direction for subsequent mHealth app development and promotion in the postepidemic era, supporting medical model reformation in China as a reference. This may provide new avenues for designing and evaluating indirect public health interventions such as health education and health promotion. Further research is needed to investigate the functions in each kind of app, which will contribute to the personalized development and specific improvement measures of mHealth apps as a health promotion strategy.

## Appendix

Multimedia Appendix 1 Supplementary tables.

Multimedia Appendix 2 Differential effect of app functions by quantile (insignificant).

Multimedia Appendix 3 Differential effect of app functions by quantile (insignificant).

## Abbreviations

CBT: cognitive behavioral therapy
mHealth: mobile health
QR: quantile regression

## Data Availability

Scientists wishing to use mobile health app study data for noncommercial purposes can obtain the data set by contacting the corresponding author.
