# Supplementary material for: Changes in Mobile Health Apps Usage Before and After the COVID-19 Outbreak in China: Semilongitudinal Survey
Source: JMIR Public Health Surveill. 2023 Feb 22;9:e40552. doi: 10.2196/40552 (PMC9996426; doi:10.2196/40552)
Supplement: Multimedia Appendix 1 [file publichealth_v9i1e40552_app1.docx]

**Table S1.** Function definitions for identifying app functionality.

| Function name | Function definition |
| --- | --- |
| Medical education | Raise health awareness and health care knowledge among the population. |
| Medical community | Communicate with peers through social networks to create a community of doctors with a professional medical atmosphere and close ties. |
| Doctor-patient communication | Both doctors and patients communicate with each other in the process of diagnosing and treating diseases to meet the health needs of patients. |
| Patient management | Provides physicians with a trackable patient management system to view and manage their outpatients. |
| Patient information management | Create patient medical records and integrate patient visit data. |
| Inquiry | Open online consultation for treatment activities, providing text, voice, and video consultation services. |
| Appointment registration | Online appointment booking service is available for patients. |
| Medical examination | Online billing services for medical examinations, test items, medical checkup programs, etc. |
| Drug purchase | Online pharmacies that conduct drug transactions. |
| Drug using | Provide drug instructions, clinical use guidelines, clinical pathways, interaction queries, etc |
| Treatment | Provide Internet diagnostic and treatment services. |
| Rehabilitation | To alleviate pain and improve the quality of life of patients by applying a combination of measures. |
| Purchase medical services | Purchase medical services online, such as medical procedure management, health insurance, etc. |
| Disease management | Create a health care plan with a variety of tailored recommendations and strategies for disease-related services to improve the condition。 |
| Bodybuilding | Sports-related, providing fitness instruction, running, cycling, dating and fitness diet guidance, etc. |
| Nutrition | Provide scientific dietary nutrition knowledge and professional nutrition skills to achieve a balanced diet and adequate nutrition. |
| Women's health | Provide women's health management, including four major areas of menstruation, pregnancy preparation, pregnancy, and childcare. |
| Men's health | Related to male health, promoting physical, psychological, and social health status in all aspects. |
| Pregnancy preparation | Provide guidance and support for health-related issues during pregnancy preparation. |
| Pregnancy | Provide health-related guidance and support during pregnancy. |
| Parenting | Provide guidance and support for health-related issues during childcare. |
| Plastic surgery | A platform for medical aesthetic services provided by aestheticians and software developers for medical aesthetic management and health care. |
| Mental health | Helping users improve their mental health through monitoring and guidance on psychological issues. |
| Cultivation of lifestyle | Provides functions of habit formation management. |
| Chinese Medicine | Allow users to study and utilize TCM-related content. |
| Vaccination | Provide vaccination-related services. |
| Test physiological indicators via mobile phone | Ability to monitor blood pressure, heart rate, and other indicators via mobile phone. |
| Genetic screening diagnosis | Provide genetic screening appointments and other services. |

**Table S2.** Research strategy for apps.

| Research Phase | Search Source | Search Terms | | | Search Criteria | Research Object | Data Collection | Number | Data Collection Time |
| --- | --- | --- | --- | --- | --- | --- | --- | --- | --- |
| Searching apps | Online shopping platform  (Tmall, JD, Pinduoduo, and Suning) | “smart sleep monitor”  “dynamic electrocardiogram” “smart blood glucose meter” “multiparameter detector”  “smart blood pressure meter” “smart health scale”  “smart bracelet” | | | Apps affiliated with the top 20 intelligent health devices for each term | - | Name | 40 | 2019.10 |
|  | iOS system  (App Store) | - | | | Top 100 or 50 apps from health-related categories |  |  | 400 | 2019.9 |
|  | Android system  (Huawei, Oppo, Vivo, Tencent, and 360) | - | | |  |  |  | 2693 | 2019.9 |
|  | Inventory of top 100 Chinese hospitals in 2018 | - | | | Apps affiliated to internet hospitals of the first 30 hospitals |  |  | 19 | 2019.10 |
|  | Baidu search engine | "mobile health app"  "medical app"  "health app"  "health applications"  "medical applications"  "health management app" | | | Apps from links in the first 20 web pages for each term |  |  | 4845 | 2019.10 |
| Mobile health app description | - | | | | | Apps that met the inclusion and exclusion criteria | Basic information | 1593 | 2019.11 |
|  |  |  |  |  |  |  | Developers | 1196 | 2019.11 |
|  |  |  |  |  |  |  | Security | 1593 | 2019.12 |
| App trials | - | | | | | Apps with all functions | Functionalities | 1285 | 2019.12-2020.2 |
| App data of postepidemic | Past search source | | - | - | | Apps that met the inclusion and exclusion criteria | Contents for epidemic | 1593 | 2021.04 |

**Table S3.** Permission listings of 1593 Android apps.

| Permission | Apps, n (%) |
| --- | --- |
| Network access | 1399 (87.8) |
| Affect the contents of users’ USB storage | 1356 (85.1) |
| Call | 797 (50.0) |
| Location | 1219 (76.5) |
| Wifi connections | 1330 (83.5) |
| Take pictures and videos | 1153 (72.4) |
| Contacts | 448 (28.1) |
| Audio | 987 (62.0) |
| Calendar | 624 (39.2) |
| Run on start-up | 894 (56.1) |
| Bluetooth | 829 (52.0) |
| Prevent phone from sleeping | 341 (21.4) |
| Additional usage request | 1105 (69.4) |
| Short messages | 287 (18.0) |
| Affect use feeling | 1041 (65.4) |
| Using fingerprint hardware | 39 ( 2.5) |
| Vibration control | 1143 (71.8) |
| Read phone status and identity | 1261 (79.2) |
| Sensor correlation | 13 ( 0.8) |
| Flash control | 487 (30.6) |
| NFC communication | 82 ( 5.2) |

**Table S4.** Rating concepts in app trials [54].

| Classification standard | App rating nouns | App rating concepts |
| --- | --- | --- |
| User  communities | Apps for health  management | Mainly applicable to the general population, providing mobile health information and mobile health management services. |
|  | Apps for medical support | Mainly applicable for patients and medical workers, providing diagnosis, treatment, and mobile medical services. |
| Mobile health service function | Medical service | Provide services for diagnosis, investigation, treatment, monitoring, and disease management. |
|  | Preventive service | Provide services promoting users’ health and following doctors’ advice. |
|  | Process service | Provide services to improve the user's health care process, such as appointment services. |
| Content of services provided by apps | Mobile health  information service | Provide medical and health-related information, including hospitals, doctors, drug information, and medical knowledge, and improve users’ health knowledge and awareness. |
|  | Mobile medical service | Provide expert registration, remote diagnosis, treatment, consultation, and localized medical services to help users solve disease problems. |
|  | Mobile health  management service | Record people's health data, formulate health plans and provide health information. |
| Tertiary prevention | Primary prevention | Cause prevention or primary prevention. Mainly takes measures against risk factors. |
|  | Secondary prevention | Early detection, early diagnosis, and early treatment. |
|  | Tertiary prevention | Symptomatic treatment. Prevent deterioration from diseases, reduce the adverse effects of the disease, and prevent recurrence and metastasis. |
| Service time | Before treatment | Determine the rating according to the point of treatment. |
|  | During treatment |  |
|  | After treatment |  |

**Table S5.** Quantile regression coefficients [95% confidence intervals] of app functions for download changes (×10^4^). (Insignificant).

| Functions | | 0.1 | 0.25 | 0.5 | 0.75 | 0.9 |
| --- | --- | --- | --- | --- | --- | --- |
| Doctor-patient communication | Coefficients | -0.0582 (-0.356, 0.239) | 0.184 (-0.594, 0.962) | 0.166 (-8.808, 9.140) | 17.051 (-30.115, 64.217) | 103.458 (-98.237, 305.153) |
|  | *P* | 0.70 | 0.64 | 0.97 | 0.48 | 0.31 |
| Inquiry | Coefficients | 0.218 (-0.0636, 0.499) | 0.0086 (-0.686, 0.703) | 0.319 (-8.298, 8.935) | -22.871 (-58.559, 12.817) | -108.276 (-277.490, 60.937) |
|  | *P* | 0.13 | 0.98 | 0.94 | 0.21 | 0.21 |
| Medical community | Coefficients | 0.500 (-0.223, 1.223) | 1.867 (-2.657, 6.390) | 9.825 (-6.164, 25.813) | 8.167 (-51.009, 67.343) | 452.477 (-471.867, 1376.821) |
|  | *P* | 0.18 | 0.42 | 0.23 | 0.79 | 0.34 |
| Patient management | Coefficients | 0.246 (-69.223, 69.715) | -0.205 (-68.358, 67.949) | -8.054 (-119.790, 103.683) | -21.872 (-801.607, 757.863) | 561.206 (-1528.844, 2651.256) |
|  | *P* | 0.99 | 1.00 | 0.89 | 0.96 | 0.60 |
| Patient information  management | Coefficients | -0.246 (-69.741, 69.249) | 0.250 (-68.829, 69.329) | 18.904 (-109.772, 147.580) | 14.253 (-760.594, 789.099) | -653.019 (-2542.748, 1236.709) |
|  | *P* | 0.99 | 0.99 | 0.77 | 0.97 | 0.50 |
| Appointment registration | Coefficients | -0.0002 (-0.113, 0.112) | 0.0449 (-0.455, 0.545) | 0.441 (-5.160, 6.042) | -15.640 (-48.026, 16.745) | -100.649 (-245.187, 43.889) |
|  | *P* | 1.00 | 0.86 | 0.88 | 0.34 | 0.17 |
| Medical examination | Coefficients | 0.0021 (-0.258, 0.262) | 0.227(-0.771, 1.224) | 1.227 (-8.516, 10.970) | -0.479 (-54.384, 53.426) | 4.713 (-815.241, 824.667) |
|  | *P* | 0.99 | 0.66 | 0.81 | 0.99 | 0.99 |
| Drug purchase | Coefficients | 3.15*10^-16^ (-0.389, 0.389) | -0.0074 (-0.957, 0.942) | 1.939 (-6.518, 10.396) | 6.464 (-319.984, 332.912) | 1491.714 (-722.306, 3705.735) |
|  | *P* | 1.00 | 0.99 | 0.65 | 0.97 | 0.19 |
| Treatment | Coefficients | 0.0005 (-0.333, 0.334) | -0.0619 (-0.799, 0.675) | -0.439 (-9.586, 8.709) | 16.173 (-67.975, 100.322) | 84.162 (-171.381, 339.705) |
|  | *P* | 1.00 | 0.87 | 0.93 | 0.71 | 0.52 |
| Nutrition | Coefficients | -0.0004 (-0.0574, 0.0566) | -0.0645 (-0.614, 0.485) | 1.992 (-5.267, 9.251) | 22.583 (-66.563, 111.730) | -45.507 (-442.897, 351.883) |
|  | *P* | 0.99 | 0.82 | 0.59 | 0.62 | 0.82 |
| Women's health | Coefficients | 0.0045 (-0.485, 0.494) | 1.376 (-2.623, 5.375) | 27.376 (-32.712, 87.464) | 124.386 (-120.448, 369.221) | 965.548 (-526.601, 2457.697) |
|  | *P* | 0.99 | 0.50 | 0.37 | 0.32 | 0.21 |
| Pregnancy | Coefficients | 0.005 (-1.915, 1.925) | -1.180(-5.506, 3.146) | -26.779 (-88.087, 34.529) | -103.803 (-375.025, 167.419) | -1052.544 (-2785.397, 680.309) |
|  | *P* | 1.00 | 0.59 | 0.39 | 0.45 | 0.23 |
| Parenting | Coefficients | -0.1642 (-1.010, 0.682) | -0.473 (-2.220, 1.275) | -1.492 (-14.327, 11.344) | 5.493 (-214.751, 225.736) | 48.561 (-1812.960, 1910.081) |
|  | *P* | 0.70 | 0.60 | 0.82 | 0.96 | 0.96 |
| Plastic surgery | Coefficients | -1.09*10^-11^ (-0.242, 0.242) | -0.079 (-2.221, 2.063) | 1.322 (-16.594, 19.238) | 72.272 (-236.017, 380.560) | 241.259 (-4301.050, 4783.568) |
|  | *P* | 1.00 | 0.94 | 0.89 | 0.65 | 0.92 |
| Mental health | Coefficients | 0.2574 (-0.153, 0.668) | 0.166 (-0.774, 1.106) | -3.078 (-10.507, 4.351) | -26.917 (-107.604, 53.771) | 18.315 (-663.435, 700.064) |
|  | *P* | 0.22 | 0.73 | 0.42 | 0.51 | 0.96 |
| Chinese medicine | Coefficients | 0.0041 (-0.191, 0.199) | -0.121 (-1.486, 1.245) | -0.260 (-12.004, 11.484) | -19.729 (-104.308, 64.851) | -47.639 (-397.789, 302.512) |
|  | *P* | 0.97 | 0.86 | 0.97 | 0.65 | 0.79 |
| Test physiological  indicators via  mobile phone | Coefficients | 0.0602 (-0.300, 0.420) | 0.396 (-0.397, 1.190) | 12.649 (-2.650, 27.947) | 91.635 (-40.430, 223.701) | 886.040 (-1010.199, 2782.279) |
|  | *P* | 0.74 | 0.33 | 0.11 | 0.17 | 0.36 |
| Genetic screening  diagnosis | Coefficients | 1.98*10^-16^ (-4.107, 4.107) | 2.470 (-9.343, 14.283) | 3.869 (-72.007, 79.744) | 57.481 (-1374.668, 1489.629) | 2428.096 (-824.865, 5681.057) |
|  | *P* | 1.00 | 0.68 | 0.92 | 0.934 | 0.14 |
| Purchase medical  Services | Coefficients | 0.0003 (-0.144, 0.144) | 0.0133 (-0.500, 0.526) | 6.948 (-4.482, 18.378) | 39.889 (-61.118, 140.895) | 223.129 (-173.474, 619.731) |
|  | *P* | 1.00 | 0.96 | 0.23 | 0.44 | 0.27 |
