# Supplementary figures and images for: Changes in Mobile Health Apps Usage Before and After the COVID-19 Outbreak in China: Semilongitudinal Survey
Source: JMIR Public Health Surveill. 2023 Feb 22;9:e40552. doi: 10.2196/40552 (PMC9996426; doi:10.2196/40552)

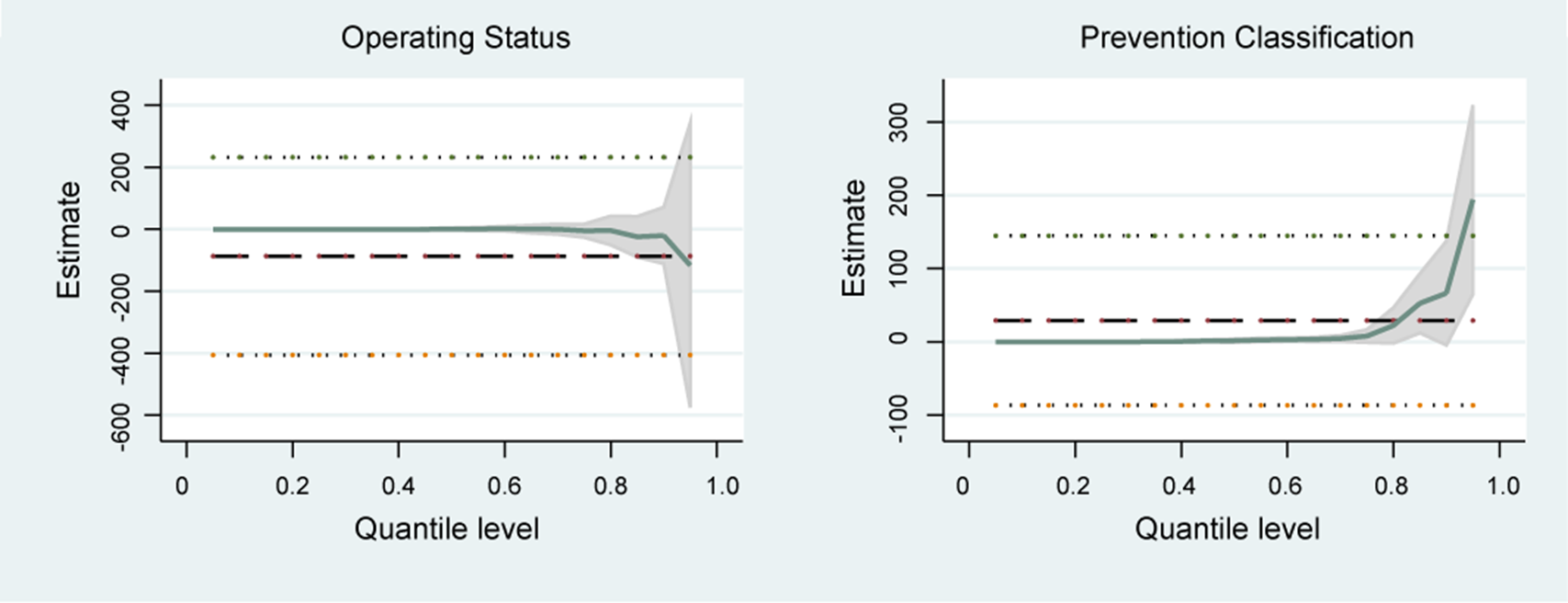

Supplement: Multimedia Appendix 2 [file publichealth_v9i1e40552_app2.png]

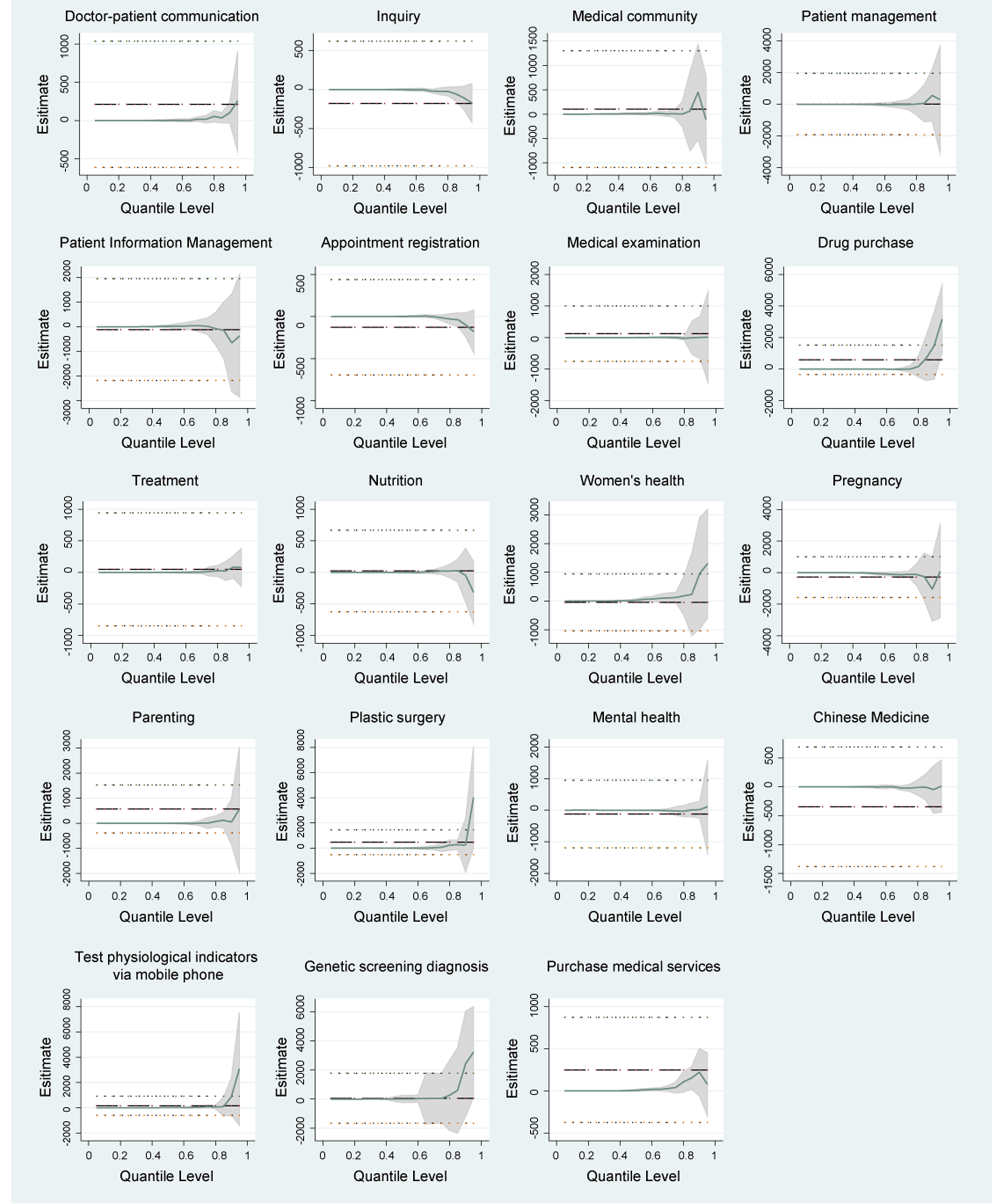

Supplement: Multimedia Appendix 3 [file publichealth_v9i1e40552_app3.png]
